# Mr. Chamberlaine, on the Amendments of the Medicine Act

**Published:** 1803-09-01

**Authors:** 


### Mr. Chamberlaine, *on the* Amendments *of the* Medicine Act.

[ Continued from pp. 165—170. ]

IT was a fortunate circumstance that these informations were laid, as it afforded to the Commissioners of the Stamps additional and striking proof of the mischiefs that, to an incalcuable extent, might befall innocent people from the introduction into the schedule of so many articles which had no right to a place there; nor would it have been very difficult to prove, what I, at a very early period of the business told them would be the case, and what I can declare to my own certain knowledge *has happened*, that many respectable apothecaries who kept retail shops, *have lost from one half to three-fourths of their retail, and others have entirely given it up through* FEAR? If then, such have been the losses to individuals in the metropolis, how great must have been the losses, on this account, when we take in the whole kingdom!

During the recess of Parliament, the Committee were employed in drawing up petitions to the Legislature, and in waiting on the different members of the House of Commons, who were accessible, for their assistance.

As it was not improbable, that certain circumstances might take place, which might render it necessary to petition parliament to be heard by counsel at the bar, and as such a step, if taken, might be attended with very considerable expence; to defray this, and various other expences of the Committee, for meetings, printing circular letters, advertisements, &c. a subscription was set on foot, and a liberal sum in the first instance subscribed.

But, although every apothecary, and every surgeon practising pharmacy, was liable to be implicated in the dangers to be dreaded from the Act, as it stood before the amendments took place, (the possibility of which danger I have clearly shewn in my former observations on the act*); yet there seemed an almost general apathy to prevail among the profession; for, of the numerous body of practising apothecaries in and within seven miles of the metropolis, there were not so many as twenty-four; and in the whole kingdom, not thirty to be found in the list of sub-

* Medical Journal, Vol. VIII. p. 127.

subscribers; and of the members of the College of Surgeons, notwithstanding so many of them keep shops, and even retail shops, not more than thirteen in all, including country practitioners.

Left as they were, to fight both the apothecaries battle and their own, it is no wonder if the druggists, who are a body of men materially interested in the sale of quack nostrums, (many individuals having concern in the exportation trade, and country orders, to the amount of from six to twenty thousand pounds per annum) should, into the body of their petitions, throw in a prayer for that most improbable of all improbable things, a total repeal of the act. Finding this object unattainable, the Committee then directed their fullest attention to the obtaining of such modifications of the act as should be best calculated to render the law as little burthensome as the nature of the case would admit of. With this view, a deputation from the Committee had several conferences with the Lord Mayor and the other members for the city of London, whose easiness of access, politeness and attention, on all occasions, so justly entitle them to the affection and praise of their fellow citizens.

When the Committee were favoured by the Minister with a printed copy of the amendments of the act of June 3d, 1802, a general meeting was called on the 23d of May last; the leading features of which were, a new schedule, and the addition of three new clauses.

I am well aware, that by entering into so minute a detail of *the history* of the Medicine Act, I shall be likely to incur the censure of some of your Readers who have nothing to do with it, for taking up so many pages. If none but medical men were readers of the Medical and Physical Journal, there would be just ground for blame; but I have some reason to know, that this Journal has a very extensive circulation among that description of persons who, above all others, are most affected by the act and is very generally read by them; I mean, *the druggists*: I therefore hope I shall stand excused by my professional brethren, if I enlarge a little on points which do not concern those who are not dealers in medicine, but materially import those who are.

I was particularly happy to see in the new schedule, that every article, which, by the desire of the gentleman who consulted me, I had struck my pen through, in the former schedule, as objectionable and improper to be in-

 serted serted, was omitted, except lozenges; and that from and after passing of the act which received the royal assent July the 4th, the apothecary, &c. is completely liberated from the restraint, which for near a twelvemonth he lay under; and may now vend, without fear of information, and without the expence of taking out a licence, all those articles of the pharmacopœia, which were unshackled previous to the passing of the late act of the 3d of June, viz. Turkey rhubarb, Indian arrow-root, Huxham's tincture of bark, Spanish juice, refined liquorice, syrup of Tolu, and several other matters enumerated in the 128th page of the 8th vol. of the Medical Journal.

Lozenges, lip-salves, issue plasters, and tooth powders had been proscribed in the lump, by the addition of the words "*of all sorts*;" but in the new schedules a modification is given, and none of these articles (lozenges excepted) are brought within the meaning of the act, unless they are sold as the invention, or secret preparation of some particular person; in that case they are liable, either with or without a printed bill or direction.

With regard to lozenges, an article of very great consumption, in the making of which, it is computed that in Great Britain only, a quantity of *refined* sugar, to the amount of not less than one hundred and seventy tons weight, is annually used, a very ample discussion took place; the issue of which discussion will appear when we come to speak of that article in our remarks on the reformed schedule.

The most regular way will be, to take the different clauses of the late act in numerical order; and collate them with those clauses in the "act to amend said act" which refer to them.

The 1st and 2d clauses remain as they were.

The first clause of the "AMENDMENT," after reciting the former act, states the expediency of repealing the schedule of said act, (of which there was great need) and of enacting another schedule in lieu thereof, and enacts that, "*from and after passing said amended act,* viz. (July 4) *said erroneous schedule, and so much of said recited act as relates thereto, shall be repealed, and the new schedule to be deemed a part of the said recited act.*

Clause 3d. *Duties to be paid by the owners and proprietors, or makers and compounders of medicines before exposure to sale; and these duties attach, not only to articles intended for home consumption, but also for EXPORTATION.*

This

This clause was not in the old act of 25 Geo. III. and was introduced on account of the defalcations in the revenue from the practices of country dealers and their town correspondents, it being known to the commissioners that large quantities of quack medicines, (such for instance as Godfrey's cordial, by the kilderkin or whole barrel) have been sent to the country, all of which has been sold in small quantities by those who, not having the fear of the Stamp Office before their eyes, have not troubled either themselves or their customers with the payment of his Majesty's duty.

However conducive it may be towards the increase of revenue to enjoin the stamping of articles in large as well as small quantities, for HOME consumption, it is a question hereafter to be decided, how far it may be policy, to charge with the *stamp duty* articles intended for EXPORTATION. Every one acquainted with finance, knows the very great repugnance all descriptions of people testify towards that particular mode of taxation. A very trifling stamp duty, attempted to be laid on in America, first laid the foundation of those discontents, which afterwards rose to so great a height, as ultimately to cause the loss of the American dominions to the crown of Great Britain.

England seems to be the grand emporium of quackery, and, poison or not poison, English quack medicines form an article of the export trade to the amount of a great many thousand pounds per annum; and of course, the consumption of articles of which they are composed, is very great; from many of which articles the revenue derives large supplies, particularly by the duties on the spirits, sugar, cochineal, gums, and various other ingredients, also on glass, paper, &c. &c. &c. used in the preparing and uttering them.

Now, if once those countries which take from Britain the largest cargoes of quack medicines, begin to shew an unwillingness or disgust at paying the additional expence of the stamps, there will not be wanting ingenious people to take advantage of that disgust, and turn it to their own account. Already have they begun to send from the continent of America, large orders to this country for the different sorts of moulded bottles, such as various quack medicines are sent abroad in, with the names of the original maker, or of the medicine itself; devices, coats of arms, &c. blown in the glass. One house only, in the drug line, in this city, exported, between the passing of the act in

June, 1802, and Christmas, three hundred gross of *empty* moulded bottles of different sorts, and, if one house alone sent out so large a consignment, to how great an amount may we not suppose the orders to have been given, when we take into consideration the very great number of houses, both druggists and glass-houses, there are in the metropolis? And for what purpose are these orders for empty bottles given? The answer is obvious. They will be filled, with imitations of British nostrums, prepared by persons employed to counterfeit them, in America and elsewhere; and the very great dealers in quack nostrums in this country, who were the persons to suggest to the minister the expediency of the measure of stamping articles for exportation, will find themselves deceived if they expect that the British stamped label will always be looked for as the infallible criterion of the genuineness of the medicine. It is far more easy to forge British stamps for quack medicines, than French assignats or American paper dollars. There are thirty-six stamps in a sheet; of course, a sheet of stamps, of the nominal value of half-a-crown each, costs the purchaser, in this country, four pounds ten shillings; whereas, in France or America, according to the price of other prints in those countries, we may suppose an engraver could afford to sell a whole sheet for sixpence or a shilling, and it would be as easy for an engraver to forge a plate of thirty-six ten-shilling stamps, as a plate of thirty-six three-halfpenny stamps; and if once this kind of traffic takes place, the revenue derived from the exportation of patent and proprietary medicines, if not entirely lost to this country, will at least suffer material diminution.

Superadded to the provisions enacted by this clause, the third clause of the "AMENDMENT" contains a new, and still stronger expedient for securing the duties; viz. "*Any person receiving from proprietors, first venders, or their agents, &c. articles subject to duty, for the purpose of selling again, without having proper stamps on each article, who shall not within ten days return the same to the person from whom such articles were received, or within the same space of time send information to the Commissioners at* SOMERSET PLACE, *and deposit such unstamped articles with the nearest* DISTRIBUTORS *of stamps, shall forfeit twenty pounds.*

The *third* clause of the AMENDMENT, being connected with the foregoing, comes properly under consideration in this place.

By

By the kindness of the Chancellor of the Exchequer, the Committee were favoured with a copy of the *new* act for amending the one so much complained of, and we were indulged with fourteen days notice to state our objections to any parts of it.

There never was an instance where the propriety of consulting persons, concerned in any particular trade or calling, before laws are made for them, was more evident, than in the *Medicine Act*. That of last year was the *third*, and still more exceptionable than the two former; which would not have been the case, had some respectable persons in the drug trade, aided by one or two well-informed apothecaries, been called into consultation, in the first instance, to contribute their advice and abilities in drawing a fair line between the preparations of the regular practitioner and the pretended arcana of charlatans and nostrum-mongers. Peculiarly fortunate was it for the gentlemen in the drug trade, who are in the habit of sending very large consignments of patent and proprietary medicines to different parts of the kingdom, and of the world, that the Committee were indulged with a sight of the new clauses before they were passed into a law.

The third clause originally stood thus. "*And be it further enacted, that upon the outside of all packages, containing* TWELVE *or more bottles, boxes, or other enclosures, sent from one dealer to another by any public conveyance, or to the Custom House for exportation, the word* "MEDICINES" *shall be written*; *and it shall be lawful for any officer of customs or excise, or of the stamp office, to open such packages, and examine if the proper labels be duly affixed to the articles*; *and, if found not duly stamped, to seize the same, &c. Officer to be rewarded for such seizure.*

By this clause, a cask or package containing three or four hundred pounds worth of drugs, and sent to an inn to go by a stage coach or waggon to the country, or to the Custom House to go by a ship, was liable to be ransacked and rummaged, if it contained so few as a dozen boxes of Scot's pills or peppermint lozenges.

This the Committee represented to the Minister; and urged, that if the clause were suffered to remain so, it would oblige them to send a servant to every inn, or to the Custom House, with every package, there to wait until an officer should come and search, in order to re-pack the goods after examination. They urged, that no officer could, or would take the pains in re-packing, necessary to

 secure secure their goods from damage, and that packing of drugs, especially such as are contained in bottles, was so nice a business, that it is not every one of their own porters who understand it properly, and that of course, the trouble, and the damage to their goods, would be incalculable.

These representations had their proper weight with Mr. Estcourt and Mr. Vansittart, and the Committee had the pleasure to find, that in the act which received the royal assent, a material alteration was made in the clause; namely, that the right of searching packages, by excise, stamp, or custom house officers, should be restricted to cases, "*wherein information on oath shall have been given before a magistrate of there being actually unstamped articles in the package, and obtaining such magistrate's warrant in writing to authorize the search.*

The fourth section or clause, which enacts that "*Duties are not to extend to articles mentioned in the book of rates, nor to unmixed drugs, sold by regular surgeons,* remains without alteration, as to words, but there is this difference in effect, that the 19th clause does not now militate against it, as before; inasmuch as several drugs, which though *unmixed,* were rendered liable to the duty, by being named in the former schedule, (such as *Turkey rhubarb, Spanish juice, arrow root,* &c.) are now omitted in the new schedule, and exempted from the stamp duty.

The 5th and 19th clauses, stand without any alteration; and a person who should content himself with only reading the marginal reference or brief statements of the contents of these clauses, might reasonably suppose that it was impossible that this act could touch any thing that was not actually a nostrum; but, LATET ANGUIS IN HERBA. We are told, Sect. 5, that "*Nothing in this act shall extend or be construed to extend, to charge with stamp duties, medicines, prepared by regular surgeons, or apothecaries, not claiming the secret, or exclusive right to the preparing the same; and not sold as patent or proprietary medicines, nor at any time* HERETOFORE, *now, or in future, shall be by any public notice, or printed paper, affixed to or delivered with such packet, held out or recommended by the proprietors, or original or first vendors thereof, as nostrums, or proprietary medicines, for the cure, relief, or prevention of complaints.*

So far, so good. No person whatever can reasonably find fault with the wording of the clauses, as far as this goes. Tax quackery with all my heart: It is but right that that they who place greater confidence in the unknown hodge podge of a stone-mason or a gingerbread-baker, than in the skill of an honest and able regular practitioner, should pay a tax *ad valorem,* over and above the price of the stuff, for their folly and credulity; but mark the concluding paragraph of each of these clauses—" *or as* SPECIFICS, *or as* BENEFICIAL FOR *the prevention, cure, or relief of any such distemper, malady, or complaint, as aforesaid.*"

Now, here every one must see, that the act goes farther than, I believe, was intended; for, if I understand this aright, it appears to mean, that not only every proprietary medicine trumpeted forth as a secret composition of great virtue by quacks and their agents; but that every medicine whatever, that ever " *was in the beginning, is now, or ever shall be,*" either publicly advertized, or sold, or dispensed, with a PRINTED DESCRIPTION of its properties, and the manner of using it, *be it* NOSTRUM *or* NO *nostrum,* must be ornamented with the quack-doctors red striped livery! This would appear as if intended as a tax upon science; because there is a possibility that there may be found particular preparations, promulgated by men of liberal principles, who would detest nothing so much as quackery, which preparations might require such cautions and instructions as to their use, as could not well be committed to memory, nor very conveniently, on account of their length, &c. given in writing. Such as these, on account of the printed instructions, it must appear very hard to include in the general mass of quack nostrums; but so I apprehend must be the case, as long as the concluding words of the clauses above alluded to, make a part of the act.

The 6th, and from thence to the 20th, relate to the rates of duties on licences and stamps; to the modes to be observed by vendors, penalties for fraudulent dealings, &c. but the rigours of the 21st, 22d, 25th, and 28th clauses of the act, are much mitigated by the mild and judicious alterations which are made by the fourth and fifth clauses of the new act, or " AMENDMENT."

For, whereas by the act of the 3d of June, the shops of medical men and druggists were laid open to the mercy of informers of every description, the 4th clause of the " AMENDMENT," enacts, that " *No person shall commence any action, or proceed before any justice in a summary way, for any penalties under the recited act, unless in the name*

*of*

*of the Attorney-General, or some persons appointed by the Commissioners of Stamps; and every action commenced by or in the name of any other person, not so authorized, are declared null and void."*

This is an important clause, inasmuch as it secures the fair dealer from the intrusion of every vagabond who either is, or pretends to be, an informer; and as the Commissioners of Stamps are gentlemen of humanity and liberality, I trust we may safely confide in the assurances communicated through their solicitor, Mr. Estcourt, that they will not prosecute on every trifling and unintentional offence against the act; and where it evidently shall appear that the party offending is innocent of every real intention of fraud, or EVASION.

And it is very necessary this clause should be made known, that all who are concerned may understand, that while the Commissioners are determined to exercise lenity on the one hand, so on the contrary they will hold the rod of correction in the other, over the *wilful* defrauder of the revenue, who will have a stronger power to contend with than the pocket of the *common* informer, as he will have the purse of the nation to combat. It may be also of use for many persons in the country, to know that none but persons acting under the jurisdiction of the stamp office have any right to molest them; as there are many wretches going about, who pretend to be informers, and who threaten timid and *law*-ignorant apothecaries, and their widows, and also shopkeepers who deal a little in drugs, with expensive law processes, whose only object is to terrify these poor people into a compliance with their exorbitant demands, and pretend to compound their sham actions for a few guineas.

[ To be continued. ]

